# Evaluating psychometric properties of the Short Form Brief Pain Inventory Sinhala Version (SF BPI-Sin) among Sinhala speaking patients with cancer pain in Sri Lanka

**DOI:** 10.1186/s40359-021-00538-1

**Published:** 2021-02-23

**Authors:** Nirosha Priyadarshani Edirisinghe, Thamasi Rekha Makuloluwa, Thamara Dilhani Amarasekara, Christine Sampatha Evangeline Goonewardena

**Affiliations:** 1 Faculty of Graduates Studies, University of Sri Jayewardenepura, Nugegoda, Sri Lanka; 2 Department of Clinical Sciences, General Sir John Kotelawala Defence University, Ratmalana, Sri Lanka; 3 Department of Nursing and Midwifery, Faculty of Allied Health Sciences, University of Sri Jayewardenepura, Nugegoda, Sri Lanka; 4 Department of Community Medicine, Faculty of Medical Sciences, University of Sri Jayewardenepura, Gangodawila, Nugegoda, Sri Lanka; 5 Cancer Research Centre, Faculty of Medical Sciences, University of Sri Jayewardenepura, Nugegoda, Sri Lanka

**Keywords:** SF BPI-sin, Cancer pain, Pain assessment, Reliability, Validity

## Abstract

**Background:**

Pain is one of the most common and unpleasant symptoms of patients with cancer. The Short Form Brief Pain Inventory (SF-BPI), has been psychometrically validated in several languages and widely used globally. Availability of a validated pain tool in Sinhala is a current requirement enabling the use among the majority of Sinhala-speaking cancer patients in Sri Lanka. The purpose of the study was to evaluate the psychometric properties of Sinhala translated version of SF BPI.

**Methods:**

The translation was done by forward–backward translation method. Content and face validity were evaluated by a panel of experts and patients with cancer pain respectively. The study included 151 participants with cancer pain, registered at the Pain Clinic, Apeksha Hospital, Sri Lanka. The reliability, discriminant and convergent validity were assessed. The confirmatory factor analysis (CFA) was conducted and evaluated the two factor (severity, interference) and three factor models (severity, affective/ activity interference). In the three factor model-1, item ‘sleep’ was included within the affective interference along with mood, relationship with others and enjoyment of life. In the three factor model-2, item ‘sleep’ was included within the activity interference along with general activities, walking and normal works. Ethical approval was obtained from the Ethics Review Committee, Faculty of Medical Sciences, University of Sri Jayewardenepura, Sri Lanka.

**Results:**

A total of 151 participants (79 males, 72 females) with a mean age of 54.6 (+/− 13.2) years were included. The composite reliability (0.902, 0.879), average variance extracted (AVE) (0.647, 0.568) and Cronbach’s alpha (0.819, 0.869) calculated for each severity and interference subscales were acceptable. The discriminant validity assessed with the heterotrait-monotrait criterion was 0.18. According to the Fornell–Larcker criterion, the square root of AVE of severity and interference factors (0.804, 0.753) greater than the correlation between the factors (0.140) demonstrated the discriminant validity. The CFA supported the three-factor model-2 (CFI—0.959, SRMR—0.0513, RMSEA—0.0699) and the values for two-factor and three-factor model-1 were marginally acceptable.

**Conclusions:**

The Sinhala version of SF BPI is a reliable and valid instrument for the assessment of cancer pain among Sinhala speaking patients in Sri Lanka.

## Background

Pain is a frequent and overwhelming symptom that diminishes the quality of life of patients with cancer [1]. More than one out of four patients with early-stage cancer experience cancer-related pain, and according to recent statistics, approximately three out of every four patients with advanced disease suffer from pain [2, 3]. Further, Oliver et al. [4] state that unrelieved pain has a significant impact on a patient’s physical, psychological, social and spiritual well-being. Studies have reported that despite the existing guidelines and new treatment modalities, a significant proportion of patients worldwide do not have satisfactory control over their cancer pain [1].

Cancer pain is complex, which varies individually in response to the associated bio-psycho-social- spiritual interactions [5]. Pain experienced in turn has a variable influence on the well-being of the individual [6]. Caraceni highlights the importance of in-depth assessment and evaluation of cancer pain as the first steps in managing cancer pain effectively [7]. Melzack and Casey suggested a three dimensional model of pain assessment comprising of sensory-discriminative, motivational-affective, and cognitive-evaluative, based on neurophysiological mechanisms of pain [8]. Therefore, the assessment of cancer pain favours the use of multidimensional scales to evaluate the overall interference of pain on the individual, apart from its severity.

The National Cancer Institute (NCI) and the Cancer Unit of World Health Organization (WHO) favour measurement tools capable of detecting the severity and impact of cancer pain and outcomes following pain interventions [9]. Among several multidimensional pain assessment tools available for the measurement of cancer-related pain in clinical and research settings [10], the short form Brief Pain Inventory (SF BPI) and McGill Pain Questionnaire are tools used globally. Under the direction of the Pain Research Group, headed by Charles S. Cleeland, the Cancer Unit of the World Health Organization (WHO) has developed the extended version of the Brief Pain Inventory in 1984 to obtain estimates of pain prevalence and, to measure the severity of pain and its interference with the function. However, currently the short version of BPI (SF BPI) is used widely as it is short, easily understood and administered to a large number of patients [11, 12]. Many studies have used SF BPI as the most beneficial multidimensional tool for the assessment of pain and its interference on patients with cancer [13, 14]. SF BPI has been recommended as a core measure by the Initiative on Methods, Measurement, and Pain Assessment in Clinical Trials (IMMPACT) [15] and Expert Working Group of the European Association of Palliative Care [7]. The SF BPI has been translated and validated into many different languages, and it has shown consistent measurement characteristics across different languages and various cultural groups [16–22]. Therefore, SF BPI has been used in multinational studies of cancer epidemiology, analgesic clinical trials, and the assessment and treatment of cancer pain. Additionally, the SF BPI has been used to derive the Pain Management Index, which is used to evaluate the adequacy of cancer pain management by comparing the intensity of pain to the standard guidelines for prescribing analgesics [23].

One of the first studies of the BPI compared the factor structure of four language versions of the BPI used to assess cancer pain in the United States, Mexico, the Philippines, and Vietnam [24]. For each language version, two factors emerged with an eigenvalue greater than one. The first factor consisted of the pain interference items and the second factor, the pain severity items. This two-factor structure was confirmed in a large national study conducted in the U.S. by the Eastern Cooperative Oncology Group [25]. Further, the study by Cleeland et al. demonstrated activity and affective subscales of the interference items using multidimensional scaling [26]. In terms of reliability, Cronbach’s alpha showed good internal consistency, ranging from 0.80 to 0.87 for the four pain severity items and, from 0.89 to 0.92 for the seven interference items by the study of Cleeland et al. [25].

In Sri Lanka, the problem of cancer is so fast-growing that it has become an essential public health concern. The Annual Health Bulletin, 2015 [27] has reported that neoplasms are ranked as the second leading cause of death in Sri Lanka since 2010. Most of these patients have advanced disease at diagnosis, putting them at much higher risk for pain and other symptoms than those with earlier-stage of disease. The number of patients suffering from cancer pain is increasing day by day, with the frequent readmissions due to unrelieved pain, with considerable impact on health care costs of the country. Among the previous cancer pain-related studies conducted in Sri Lanka, none have used multidimensional pain scales to assess the overall impact of the cancer pain on the patients. Except for the validated Sinhala version of Short-form McGill Pain Questionaire-2, which mainly measures the qualities of pain [28], no other Sinhala versions of validated multidimensional instrument is currently available to assess cancer pain in Sri Lanka. ‘Sinhala’, being the native language of the majority of the Sri Lankan population, the availability of validated pain scales in the Sinhala language therefore immensely supports the facilitation of the assessment of pain and its interference on functions among Sinhala speaking patients with cancer. The Sinhala version of SF-BPI (SF BPI-Sin) meets these requirements.

This study was conducted to translate, culturally adapt, and validate the SF BPI, enabling the assessment of pain severity and interference among Sinhala speaking patients with cancer pain. The psychometric properties of SF BPI-Sin in terms of face, content, consensual validity, construct validity and reliability were evaluated.

## Methods

### Study design and setting of the study

The validation study includes translation, cultural adaptation, and evaluation of psychometric properties. The translation was carried out using the widely accepted forward–backward translation method, and in consultation with the clinical and subject experts in the field of pain medicine. A cross-sectional study was conducted to evaluate the psychometric properties among Sinhala speaking patients with cancer pain, registered at the Pain Clinic of Apeksha Hospital, Maharagama, Sri Lanka.

### The procedure of translation of SF BPI English version to Sinhala version

The translation process was conducted according to the guidelines provided by the MD Anderson Cancer Center, USA. The translation process consists of 5 steps: forward translation, back translation, review by experts, cognitive debriefing, and proofreading. Two sets of forward translations in-to Sinhalese language, back translations into English, expert opinions, and cognitive debriefings were carried out separately. Two preliminary Sinhala versions were evaluated and a harmonized Sinhala version was prepared. The forward translated harmonized version was then back-translated by two independent translators who speak both Sinhala and English languages fluently. For the items or response choices where back-translations and original versions did not match, the choice of words was discussed among the translators until a final version was reconciled under the guidance of the original authors. Content validity was checked by obtaining expert opinion. Experts who were invited comprised of Consultant Anesthetists specialized in pain medicine and senior academics in nursing, Consultant Community Physicians and nurses in charge of pain clinics. The expert panel assessed the content, the appropriateness of the words used and their cultural relevance and the translation equivalence of each item. The resulting version was used in cognitive debriefings of 15 patients with cancer pain to evaluate the face validity. Participants were chosen purposively (Heterogeneous Sampling) by looking at subjects from all available angles to ensure selection of a diverse group of patients across a broad spectrum of socio-demographic characteristics, representing different provinces in the country with different types of cancers. The interviews were directed towards each item in the questionnaire separately, in order to determine if the word flow in the questions has made any of the items difficult to understand, confusing, difficult to answer and objectionable. It was also examined whether the questions could have been asked in a different way to improve comprehensibility. Based on the comments of the participants, minor changes were made to the document as a collective decision of the research team, without affecting the meaning of the items. Face validity testing needed no further major revisions in respect of the item content or scoring. Proofreading was carried out to eliminate any grammatical, spelling, typographical and/or formatting errors.

### Sample and sample size calculation

The study included all patients above the age of 18 years diagnosed with any type of cancer, with a pain score > 1 on Numerical Rating Scale, with pain related to primary lesion, secondaries, radiotherapy, or chemotherapy, and whose pain lasted at least for three months or more since the diagnosis of cancer. Irrespective of ethnicity, all patients capable of speaking and comprehending Sinhala language were included in the study. Patients whose pain is due to any cause other than cancer, or pains that lasted less than three months from the time of assessment, or who are too frail or mentally unfit or unwilling to give informed consent were excluded from the study.

The sample size was calculated considering the rule of thumb of 5–10 subjects per item [29]. There were 15 items in the instrument and the calculated sample size was 150, considering 10:1 subject to the variable ratio. Five percent was added considering non response rate; therefore, the final calculated sample size was 158. Participants fulfilling the selection criteria were recruited for the validation study by consecutive sampling method until the sample size was reached.

Out of all the data sheets received, five were incomplete and two were noted as outliers. Therefore, data from 151 participants were included for analysis (response rate—95.5%). The mean age of the participants was 54.6 (+/− 13.2), and the age range varied from 20 to 80 years. The majority, 52.3% were males (n = 79) and 47.7% were females (n = 72). Among the participants, majority (25.2%) were diagnosed with uro-genital cancers (n = 38) followed by gastro-intestinal (n = 36, 23.8%) and oro-facial (n = 31, 20.5%). The mean duration of pain, since its onset, was 8.14 (+/−  10.5) months. Table 1 depicts the socio-demographic characteristics of the sample.

**Table 1:** Socio-demographic characteristics (n = 151)

	Frequency	Percentage
Age		
Mean 54.6 (SD ± 13.2) Range 20–80		
Gender		
Male	79	52.3
Female	72	47.7
Race		
Sinhala	133	88
Tamil	10	6.6
Muslim	3	2
Burger	4	2.6
Malay	1	0.7
Level of education		
Not attended to school	11	7.2
Up to grade 5	54	35.7
Up to grade 11	50	33.1
Up to grade 13	33	21.9
Graduate	2	1.3
Post graduate	1	0.7
Marital status		
Single	13	8.6
Married	111	73.5
Divorced	4	2.6
Living together	1	0.7
Widow	22	14.6
Cancer diagnosis		
Uro-genital	38	25.2
Gastro-intestinal	36	23.8
Oro-facial	31	20.5
Other	17	11.3
Lung	14	9.3
Breast	9	6
Blood	6	4

### Measurements

SF-BPI-Sinhala is a self-administered or interviewer-administered pain rating scale with four questions related to pain intensity, with responses rated on a numerical rating scale ranging from 0 to 10; “0” is “no pain” and “10” is “pain as bad (excruciating) as you can imagine”. Cancer patients rate their worst, least and average pain in the last 24 h and, the pain experienced at the time they were responding to the questionnaire. Pain interference on general activity, mood, walking ability, normal work, relationships with other people, sleep and enjoyment of life were rated on a numerical scale from 0 = “Does not interfere” to 10 = “Interferes completely”. The scale comprised of a diagram of a human figure for locating areas of pain and, questions about pain medications and the percentages of pain relief achieved with medications in the last 24 h.

### Procedure of data collection

The data were collected between November 2017 and May 2018. Those who expressed interest in participating in fulfilling the inclusion criteria were invited to read and sign a consent form. After obtaining the informed written consent, data were collected from the participants of the validation study using an interviewer-administered SF-BPI-Sin questionnaire uniformly by the principal investigator. Additionally, the information regarding the demographic characteristics, type of cancer, duration of pain since the diagnosis of cancer, other comorbidities, and the details of the previous and current treatment interventions were noted.

### Analysis

Statistical analysis was conducted using IBM SPSS Statistics version 20.0 for windows [30]. Internal consistency was assessed using composite reliability (CR), (range from 0.70 to 0.90) [31] and Cronbach’s alpha coefficients (≥ 0.70) as reliability measures [32]. Construct validity was evaluated using convergent and divergent validity and confirmatory factor analysis (CFA). The measurement model was assessed using the average variance extracted (AVE) (> 0.5) and the composite reliability (CR). Discriminant validity was examined by Fornell Larcker Criterion; in which the square root of each construct’s AVE should have a greater value than the correlations with other latent constructs [33] and heterotrait-monotrait (HTMT) criterion (< 0.9) [34]. After ensuring the reliability and validity, CFA of the construct measures was performed using Lisrel 10.20 for windows [35], with the following fit indices. Chi-square value (low-value) and the associated degrees of freedom, Comparative Fit Index (CFI) > 0.95, Standardized Root Mean Square Residual (SRMR) < 0.08 and Root Mean Square Error of Approximation (RMSEA) < 0.06 [36]. We hypothesized the original two-factor model (severity, interference) and three-factor model-1, as found in the factor models in the literature (severity, activity interference—own general activities, walking, normal works and affective interference- mood, relationship with others, enjoyment of life, sleep), and three-factor model-2 as alternatively proposed by the original test authors (severity, activity interference—own general activities, walking, normal works, sleep and affective interference—mood, relationship with others, enjoyment of life).

### Ethical considerations

The study was approved by the Ethics Review Committee (ERC No: 32/17) of the Faculty of Medical Sciences, University of Sri Jayewardenepura, Sri Lanka. Informed written consent was obtained from the study participants before the collection of data. Administrative permission for data collection was obtained from the Director, Apeksha Hospital Maharagama, Sri Lanka.

## Results

Prior to performing analysis, the data set was assessed for quality, suitability, and any missing data or violations of the assumptions demanded by the analytical techniques in CFA. Respondents with two or more missing items were excluded from the analysis. Continuous variables were descriptively summarized using means and standard deviations. Standardized values (Z-scores) ± 3 were assessed to identify the univariate outliers of each item. Two outliers were identified with a Z-score of 3.4. The raw data set was checked for errors in data entry. In the absence of errors and major deviations in the data set, no adjustments were made. Data were assessed for multivariate outliers using Mahalanobis Distance and, two were detected and removed. The normalcy of data assessed using histograms and Q–Q plots showed several items with non-normal distributions. Both the Kolmogorov–Smirnov test and the Shapiro–Wilk’s tests were significant (p < 0.05), indicating that the data were not normally distributed. Standardized skewness and kurtosis values were calculated; seven and five items out of 11 items had exceeding ± 3 values indicating high skewness and kurtosis respectively. The sampling adequacy was assessed using the Kaiser–Meyer–Olkin measure of sampling adequacy (desirable value > 0.8) 0.816 and Bartlett’s test of sphericity was noted to be significant [37].

### Descriptive analysis of the SF BPI-Sin

Among the study participants, worst, least, average and current pain had mean values (SD) of 8.06 (1.79), 1.11 (1.37), 4.75 (1.54) and 3.87 (1.81) respectively. Concerning the interference, pain interfered mostly with the enjoyment of life with mean values (SD) 7.87 (2.13), mood 7.70 (2.21), normal works 6.93 (2.46), and sleep 6.17 (2.62). The least interference was observed on relationships with others with a mean of 4.58 (2.34) as illustrated in Table 2.

**Table 2:** Descriptive statistics of SF BPI-Sin scores (n = 151)

BPI Item	Mean	SD	Minimum	Maximum	Skewness	Kurtosis
Worst pain	8.06	1.79	3	10	− 0.522	− 0.780
Least pain	1.11	1.37	0	6	1.233	1.245
Average pain	4.75	1.54	1	8	− 0.168	− 0.410
Pain now	3.87	1.81	0	8	− 0.330	− 0.351
General activity	5.59	2.57	0	10	− 0.507	− 0.545
Walking ability	5.23	3.24	0	10	− 0.320	− 1.293
Normal works	6.93	2.46	0	10	− 1.122	0.698
Sleep	6.17	2.62	0	10	− 1.115	0.582
Relationships	4.58	2.34	0	10	0.295	− 0.527
Enjoyment of life	7.87	2.13	0	10	− 1.813	4.060
Mood	7.70	2.21	0	10	− 1.693	2.993

### Psychometric properties

Assessment of the reliability was conducted by CR and Chronbach's alpha. The CR was 0.902 and 0.879 and, the Chronbach's alpha values were found to be 0.819 and 0.868 for severity and interference subscales respectively. Further, Cronbach’s alpha could be increased by 0.020, if item ‘least pain’ deleted, as mentioned in Table 3.

**Table 3:** Comparison of internal consistency among subscales (n = 151)

BPI Items	Cronbach’s alpha					
	Two factors		Three factor 1		Three factor 2	
	Item total correlation	Alpha if item deleted	Item total correlation	Alpha if item deleted	Item total correlation	Alpha if item deleted
	Severity (alpha = 0.819)		Severity (alpha = 0.819)		Severity (alpha = 0.819)	
Worst pain	0.637	0.776	0.637	0.776	0.637	0.776
Least pain	0.477	**0.839**	0.477	**0.839**	0.477	**0.839**
Average pain	0.781	0.710	0.781	0.710	0.781	0.710
Pain now	0.698	0.745	0.698	0.745	0.698	0.745

	Interference (alpha = 0.868)		Activity (alpha = 0.829)		Activity (alpha = 0.813)	
General activity	0.664	0.847	0.644	0.803	0.645	0.760
Walking ability	0.631	0.857	0.669	0.806	0.659	0.759
Normal works	0.796	0.829	0.784	0.683	0.777	0.704

			Affective (alpha = 0.789)			
Sleep	0.571	0.859	0.567	0.759	0.487	**0.828**

					Affective (alpha = 0.759)	
Relationships with others	0.534	0.863	0.476	**0.796**	0.492	**0.790**
Enjoyment of life	0.680	0.847	0.713	0.685	0.665	0.593
Mood	0.691	0.845	0.664	0.706	0. 621	0.640

The items which increase the alpha values ‘if deleted,’ more than the subscale values were indicated with bold numerals

The AVE values used to assess convergent validity of severity and interference subscales were 0.647 and 0.568 as shown in Table 4. Discriminant validity was examined according to the Fornell-Larcker criterion which shows the square root of AVE for severity and interference subscales as 0.80 and 0.753 respectively. According to the component correlation matrix, the correlation between the factors was 0.140. Discriminant validity assessed by HTMT was 0.18, which is lower than the threshold, and which enabled concluding the discriminant validity of SF BPI-Sin. Item-to-total correlations were measured to test how well each item score correlates with the overall SF BPI – Sin score. The item-to-total correlations were above the acceptable level > 0.50 [31] except for ‘least pain’ (0.477). All the inter-item correlations were observed to be within the acceptable range (0.30 to 0.80) [31]; from 0.330 to 0.697.

**Table 4:** Composite reliability (CR), the square root of the Average Variance Extracted (AVE) (in bold) and correlations between constructs (off-diagonal)

Latent constructs	Composite reliability	Average variance extracted	Latent constructs	
			Severity subscale	Interference subscale
Severity subscale	0.902	0.647	**0.804**	
Interference subscale	0.879	0.568	0.140	**0.753**

CFA was performed for the SF BPI items, after ensuring that the required assumptions had been met. The robust maximum likelihood method was adjusted for the non-normality of the data, as recommended by LISREL software, to estimate the model parameters [38]. The 3 factor model-2 came up with better model fit indices according to the combinational rule by Hu and Bentler, [36] and recommendations by Jackson et al. [39] as shown in Table 5. Accordingly, authors used Chi-squared value of 71.24 (df = 41) (p < 0.000), CFI (0.959) in combination with SRMR (0.0513) and RMSEA = 0.0699 to evaluate the model fit. The other fit indices considered were as follows; GFI = 0.922, AGFI = 0.874 NNFI = 0.944, PGFI = 0.573 and PNFI = 0.678 [36, 38–40].

**Table 5:** Fit Indices for Confirmatory Factor Models of the sample (n = 151)

Indices	Reference values	2 Factor model	3 Factor model-1	3 Factor model- 2
Chi-square		91.53	86.86	71.24
Df		43	41	41
P		< 0.05	< 0.05	< 0.05
SRMR	< 0.08	0.0594	0.0544	0.0513
CFI	> 0.95	0.933	0.946	0.959
RMSEA (90%CI)	< 0.06	0.0864 (0.0618; 0.111)	0.0861 (0.0608; 0.111)	0.0699 (0.0415; 0.0965)
GFI	> 0.9	0.898	0.911	0.922
AGFI	> 0.9	0.844	0.857	0.874
NNFI	> 0.95	0.915	0.928	0.944
PGFI	> 0.5	0.585	0.566	0.573
PNFI	> 0.5	0.691	0.674	0.678

*RMSEA* Root Mean Square Error of Approximation, *GFI* Goodness of Fit Index, *AGFI* Adjusted Goodness of Fit Index, *SRMR* Standardized Root Mean Square Residual, *CFI* Comparative Fit Index, *NNFI* Non-Normed Fit Index, *PGFI* Parsimony Goodness of Fit Index, *PNF* Parsimonious Normed Fit Index

## Discussion

The SF BPI, is one of the most widely used multidimensional pain scales, with items for pain severity assessment and for measuring pain interference. The main objective of the present study was to determine the validity and reliability of the translated pain tool, SF BPI-Sin. The SF BPI questionnaire itself is short and simple and it consists of a relatively minimal number of descriptive words that have been translated and validated in several languages. The conceptual equivalence between the original and the Sinhalese version of the SF BPI was well maintained through the approach of forward and backward translation. Content validity was confirmed as acceptable by the experts in the field of pain, and the face validity was confirmed by the patients. Further, it was demonstrated to be a convenient and user-friendly tool. Apeksha Hospital Maharagama, the premier treatment center for patients with cancers in Sri Lanka, was chosen as the study setting for sampling, due to the ethno-geographic and cultural diversity of its patients. The results of this study can be generalized to a wider population of patients experiencing cancer pain in Sri Lanka.

The current study has demonstrated the psychometric properties of the SF BPI-Sin, and hence, it can be used as a valid and reliable tool that measure of cancer pain and its interference on the functions of Sinhala speaking patients during the clinical practice and in a research setting. Pain outcome measures such as ‘pain worst’ and ‘pain average’ would be useful in evaluating the response to pain interventions used in the preceding 24 h. The mean value of the SF BPI-Sin’s interference items would be a useful overall measure of the impact of pain on functions.

Several studies conducted on the psychometric properties of the SF BPI, translated and validated in different languages, have shown SF BPI as a valid and reliable scale to assess the pain of patients with cancers [17, 22, 41]. Apart from the two factor model, the three-factor model was also validated among patients with cancers and other chronic pain conditions [42].

The reliability was assessed by internal consistency; with CR, and Cronbach’s alpha. All the CR values were within the acceptable range, and alpha coefficients were above the acceptable threshold of 0.7. The CR is the upper bound for internal consistency and it was identified to be strong in this study, demonstrating acceptable internal consistency of the scales. Although there is a slight increase in ‘if item deleted’, Cronbach’s alpha values for ‘least pain’, authors and experts in the field decided to retain all the items in the instrument, considering its strong CR and having considered its worthiness in pain assessment. However, the results demonstrated reasonably high levels of internal consistency and CR even without removing the item. The study conducted by Zeng [43] has shown the psychometric validity and reliability with the three-factor model, after removing ‘least pain’ and ‘sleep’ items. Nevertheless, ten out of eleven corrected item-total correlations were more than 0.5, indicating that no item should be revised or excluded [44]. The inter-item correlations and corrected item-total correlations of this study further support the acceptable reliability of SF BPI-Sin.

The construct validity of the scale was assessed by convergent validity, divergent validity and CFA. Convergent validity was assessed by AVE and CR. The AVE calculated for each construct was above the acceptable level of 0.5 for the two-factor model, and CR was within the acceptable range. The discriminant validity assessed with Fornell-Larcker criterion; which was commonly used to assess the degree of shared variance between the latent variables of the model. The square root of AVE of each two constructs was greater than the correlation involving the constructs that demonstrate acceptable discriminant validity. Further, the discriminated validity was assessed by a new and more stringent method of HTMT criterion, and it demonstrated acceptable discriminating ability of two constructs.

Authors have evaluated the construct validity of the tool with CFA, for originally hypothesized two-factor model, alternatively suggested, three-factor model-1 and three-factor model-2. The best combinational rule fit indices of CFI and SRMR were used for minimizing error rates due to the small (< 250) sample size of this study. The three-factor model-2 exhibited well-fitted values compared to the two-factor model. However, the hypothesized two factor model and three factor model-1 also demonstrated marginally acceptable fit indices. In the literature, several interpretations were observed for the inclusion or exclusion of item ‘sleep’ in factor models. Cleeland et al. [24] have suggested that the item ‘sleep’ would be closely related to the activity interference, while Klepstad et al. [16] and Atkinson et al. [45] proposed that ‘sleep’ be part of affective interference. Saxena et al. [46] recommend the sleep item be removed from the instrument. Zeng and Wu et al. [42, 43] have removed the item sleep from the instrument. Disturbed ‘sleep’ is one of the main complaints made by the patients with cancer pain. The studies have shown that sleep disturbances contributed to increased pain or pain interfered with sleep [47]. Further, it has been suggested that sleep disturbances in chronic pain patients may increase pain sensitivity and create a self-perpetuating cycle of sleep disruption and increased pain [48]. As authors, we strongly believe that the interference to sleep item should be assessed among patients with cancer pain, irrespective of the factor model it belongs to. Comparison of the factor models of the original study and other studies identified in literature was illustrated in Table 6.

**Table 6:** Factor models of SF BPI in original study and other studies

Factors	Two factors			Three factors without item ‘sleep’			Three factor-1		Three factor-2
	Original study [11]	Aisyaturridha et al. [41]	Ger et al. [21]	Saxena et al. [46]	Zeng et al. [43]	Wu et al. [42]	Klepstad et al. [17]	Atkinson et al. [45]	Cleeland et al. [24]
Severity	WLAN	WLAN	WLAN	WLAN	WLAN	WLAN	WLAN	WLAN	WLAN
Activity	WAW, sleep, REM	WAW, sleep, REM	WAW, sleep, REM	WAW	WAW	WAW	WAW	WAW	WAW, sleep
Affective				REM	REM	REM	REM, sleep	REM, sleep	REM

WLAN—the pain severity items (W—worst, L—least, A—average, N—now)

REM—the affective cluster of interference items (R—relations with others, E—enjoyment of life and M—mood),

WAW—the activity cluster of interference items (W—walking, A—general activity and W—work)

Findings of this study support the use of either the two-factor or three-factor model-2 for scoring the SF BPI-Sin. Our observations are comparable with a number of previous studies in which the two factor model (severity and interference) of the SF BPI (27, 41). In populations where a differentiation between activity and affective interference is not appropriate or practicable, the two-factor structure can be used. However, when an additional distinction is required between activity and affective interference, the three-factor model-2 would be realistic and acceptable for use.

The present study has a few limitations. Firstly, we have not evaluated the SF BPI-Sin's test–retest reliability and responsiveness. As a patient reported outcome instrument, SF BPI-Sin needs to achieve the standards of validity and reliability. Among reliability analyses, one of the most important measure is the test–retest reliability; which is a measure of the reproducibility of the scale and the ability to provide consistent scores over time in a stable population. A basic concept with regard to test–retest reliability is the need to re-test among clinically stable patients [49]. This situation is particularly challenging when it comes to patients with cancer, because of their faster clinical deterioration. The retest of a clinically unstable patient may incorrectly define the tool as a non-reliable tool. However, we believe that the translated tool would be useful in comprehensive assessment of severity and interference of cancer pain and, in monitoring the response to pain interventions. Secondly, few previous studies discuss on the relationship between severity of pain with the type, stage of cancer and the type of treatment intervention [50]. Although, the present study population was not limited to a specific type of cancers, further studies are recommended using patients with different types of cancers, to check conformity with our results. Further, it would have been ideal if the concurrent validity was assessed at the time of conducting this study using a gold standard instrument to measure cancer pain.

## Conclusions

We conclude that SF BPI-Sin is a statistically confirmed, valid and reliable tool that can be used to assess the intensity of cancer pain and its interference with the functions of the Sinhala speaking population in Sri Lanka during the routine clinical practice and in clinical research.

## Data Availability

The data utilized to support the findings of this study are available from the corresponding author upon reasonable request.

## Abbreviations

SF BPI-Sin: Short form Brief Pain Inventory – Sinhala version
CFA: Confirmatory Factor Analysis
RMSEA: Root Mean Square Error of Approximation
GFI: Goodness of Fit Index
AGFI: Adjusted Goodness of Fit Index
SRMR: Standardized Root Mean Square Residual
CFI: Comparative Fit Index
NNFI: Non-Normed Fit Index
PGFI: Parsimony Goodness of Fit Index
PNFI: Parsimonious Normed Fit Index
HTMT: Heterotrait-Monotrait criterion
AVE: Average Variance Extracted
CR: Composite Reliability
